# Efficient removal of tetracycline from aqueous solution by K_2_CO_3_ activated penicillin fermentation residue biochar

**DOI:** 10.3389/fchem.2022.1078877

**Published:** 2022-12-13

**Authors:** Yanfang Liu, Wei Gao, Sijie Yin, Rui Liu, Zaixing Li

**Affiliations:** ^1^ School of Environmental Science and Engineering, Hebei University of Science and Technology, Shijiazhuang, China; ^2^ Pollution Prevention Biotechnology Laboratory of Hebei Province, Shijiazhuang, China; ^3^ School of Civil Engineering, Hebei University of Science and Technology, Shijiazhuang, China

**Keywords:** tetracycline, biochar, adsorption, K_2_CO_3_ activating, penicillin fermentation residue

## Abstract

In this study, biochar was prepared using penicillin fermentation residue (PR) as the raw material by different methods. The adsorption behavior and adsorption mechanism of biochar on tetracycline (TC) in an aqueous environment were investigated. The results showed that K_2_CO_3_ as an activator could effectively make porous structures, and that biochar with mesoporous or microporous could be prepared in a controlled manner with two kinds of different activation methods, the dry mixing method and the impregnation method. The dry mixing method could create more mesopores, while the impregnation method could prepare more micropores. Microporous biochar (IKBCH) with a high specific surface area could be prepared by the impregnation method combined with HCl soaking, which has an excellent adsorption effect on tetracycline. When the concentration of tetracycline was 200 mg/L, the removal rate of 99.91% could be achieved with the dosage of microporous biochar at 1 g/L. The adsorption process was in accordance with the Langmuir model and the pseudo-second-order model, respectively. The maximum adsorption capacity of IKBCH was 268.55 mg/g (25°C). The adsorption mechanisms were pore filling, π-π interaction, electrostatic adsorption, and hydrogen bond. Its stable and wide applicability adsorption process does not cause ecological pollution in the aqueous environment, and it is a promising biochar adsorbent.

## 1 Introduction

Tetracycline (TC) is a broad-spectrum antibiotic that is widely used in the treatment of diseases and as a feed additive to promote the growth of animals (Hu et al., 2022). However, relevant studies have shown that 70%–90% of TC was discharged directly or indirectly into the ecosystem in the form of active pharmaceutical ingredients, especially into water, due to its structural stability and resistance to degradation (Liu et al., 2021; He et al., 2022). TC may re-enter the food chain through water resources, thus threatening animal and human health (Zhao et al., 2021). In addition, TC pollution can cause the local production of antibiotic resistance genes, destroy the aquatic microbial community, and harm the local ecological environment (Li et al., 2022). Therefore, efficient and reliable TC removal technology is in urgent need of development.

Penicillin fermentation residue (PR) is the residue produced during the penicillin preparation process, which is classified as hazardous solid waste in China (Hong et al., 2021), and is also a kind of biomass that needs to be urgently treated because of environmental pollution and the generation of antibiotic resistance genes (Ren et al., 2021). PR is rich in organic matter and considered one of the excellent raw materials for preparing biochar by pyrolysis (Wei et al., 2022). It has been reported that biochar with a high specific surface area was prepared by the pyrolysis of PR (Wang et al., 2021a), while the harmless treatment of PR was achieved (Wang et al., 2021b). The preparation of biochar by pyrolysis is considered as a potential technology.

Biochar, a kind of carbonaceous material with a high specific surface area, showed great potential for application because of its wide availability of raw materials, low cost, and good physical/chemical surface characteristics (Dai et al., 2020a). Biochar can be made from organic biomass (Shan et al., 2020), such as straw, rice husk, sewage sludge, egg shell, *etc.* Dai et al. (2020b) prepared rice straw derived biochar, and the adsorption capacity for TC reached 98.33 mg/g, showing good wastewater treatment and straw resource reuse potential. Wang et al. (2020) prepared biochar from bamboo by the steam activation method, which showed excellent performance for the synergistic removal of Cu^2+^ and TC. Ma et al. (2021) activated municipal sludge with zinc chloride and prepared biochar to remove TC and ciprofloxacin (CIP) from wastewater. They found the maximum adsorption capacity of the biochar for TC and CIP was 145 mg/g and 74.2 mg/g, respectively. It can simultaneously achieve the utilization of sludge resources and antibiotic removal. The traditional biochar produced from common solid wastes showed wide application value while for dangerous solid wastes such as PR, the feasibility of preparing biochar to adsorb pollutants in water needs further study.

In this study, PR derived biochar was prepared and used for the adsorption of TC, a typical antibiotic in water. The following objectives were pursued: 1) to analyze the components of PR and characterize the biochar; 2) to systematically explore the effects of different experimental factors, such as biochar dosage, solution pH value, ionic strength, reaction time, initial concentration, and temperature, on the adsorption of TC; 3) to evaluate the desorption performance and the recyclability of the biochar; 4) to investigate the mechanisms of TC adsorption by the biochar; 5) to evaluate the environmental impact and economy of the biochar.

## 2 Materials and methods

### 2.1 Materials

PR was taken from a pharmaceutical factory in Shijiazhuang (Shijiazhuang, China). To prepare for further use, it was dried in an oven for 24 h at 80°C, then crushed and sieved through an 80-mesh sieve. TC (C_22_H_24_N_2_O_8_) was bought from Shanghai Macklin Co. (Shanghai, China). Other chemicals such as NaOH, HCl, H_2_O_2_, K_2_CO_3_, NaNO_3_, Na_2_CO_3_, Na_2_SO_4_, and NaCl were purchased from Tianjin Yongda Chemical Reagent Co. (Tianjin, China). The purity of the purchased chemical reagents was all analytical purity, and deionized water was used to prepare all solutions.

### 2.2 Biochar production

Two different activation methods were used to activate biochar: 1) impregnation activation method, and 2) dry mixing method. Three routes were used to prepare biochar: 1) direct pyrolysis, 2) activated-pyrolysis, and 3) activated-pyrolysis-acid soaking.

The detailed activation methods were as follows: 1) impregnation activation method: the equal mass ratio of PR and K_2_CO_3_ was mixed and transferred to a beaker, and then 200 ml of deionized water was added and stirred for 2 h. The mixture was separated by centrifugation and placed in an oven, where it was dried at 105°C to obtain pre-activated dry penicillin fermentation residue (IPR). 2) Dry mixing method: The equal mass ratio of PR and K_2_CO_3_ was mixed directly and thoroughly to obtain the pre-activated dry penicillin fermentation residue (DPR).

The detailed preparation methods were as follows: 1) Direct pyrolysis: PR was placed in a tube furnace, heated to 600°C at a rate of 10°C/min with nitrogen as a protective gas, and then kept for 2 h to obtain biochar (PRBC). 2) Activated-pyrolysis: IPR or DPR were put in a tube furnace and pyrolyzed under the same conditions to obtain activated biochar (IKBC or DKBC), respectively. 3) Activated-pyrolysis-acid soaking method: IKBC or DKBC were soaked in 3 mol/L HCl for 1 h to fully remove excess K_2_CO_3_ and inorganic impurities, then washed repeatedly with deionized water to neutrality and dried at 105°C to obtain activated acid-soaked biochar (IKBCH or DKBCH), respectively.

### 2.3 Characterizations

The following measurements were operated for characterization the physico-chemical property of PR. The elemental content (C, N, S, and H) was analyzed using an elemental analyzer (Elemantar Vario EL cube, Germany). The difference was used to calculate the oxygen content. Thermoanalyzer systems (Rigaku TG-DTA 8122, Japan) were used to analyze the thermal stability. An industrial analyzer (SX2-10-12N, China) was used to determine the content of moisture, ash, and volatile matter in PR, and the difference was used to calculate the fixed carbon content. In the Supplementary Text S1, the component analysis of PR was introduced in detail, and the contents of crude protein, crude fat, and total sugar in the residue were also determined.

A series of measurements was conducted to characterize the physical structure of biochar. The BET and pore structure characteristics were measured by ASAP 2460 (Micromeritics ASAP 2460, United States). The surface morphology and structure were observed by SEM (TESCAN MIRA4, Czech Republic). The functional groups were confirmed by FT-IR (Thermo Scientific Nicolet iS20, United States) in the wavenumber range of 500–4,000 cm^−1^. The chemical bond environment and adsorption sites analysis was taken by an X-ray photoelectron spectroscopy (Thermo Fisher ESCALAB XI+, United States) with the monochromatic Al Kα X-ray radiation. A laster confocal Raman scattering spectrometer (Thermo Scientific DXR Raman Microscope, United States) was used to measure the Raman spectra of the samples. The pH_pzc_ was measured according to the method described by Jung (Jung et al., 2017). Determination of antibiotic residues in biochar by high performance liquid chromatography (HPLC), details in Supplementary Text S1.

### 2.4 Batch experiments

The effects of different conditions for reaction were examined in adsorption experiments. A TC solution of 200 mg/L (pH about 6.5) was prepared by dissolving tetracycline in volumetric flasks. The adsorption experiments were carried out in a flask, unless otherwise specified, the flasks were shaken at 25°C at 200 rpm for 2 h. To study the effect of biochar dosage on TC removal, 0.02, 0.04, 0.06, 0.08, and 0.1 g biochar were added into 40 ml TC solution (0.5–2.5 g/L). The TC adsorption capacity of biochar under different pH (2, 4, 6, 8, 10, and 12) conditions was studied. 0.1 mol/L HCl or NaOH was used to adjust the pH of the solution at the biochar dosage of 0.6 g/L. Various concentrations (0, 10, 100 mmol/L) of KCl, NaCl, CaCl_2_, or MgCl_2_ were used to investigate the effect of ions on TC adsorption, respectively, under the biochar dosage of 0.75 g/L. Adsorption isotherms were determined for different initial concentrations (10, 25, 50, 100, 150, and 200 mg/L) and different temperatures (25, 35, and 45°C) under the biochar dosage of 0.5 g/L. For the analysis of TC adsorption kinetics, the flask was shaken for different times (1, 5, 10, 20, 40, 60, and 120 min) at the biochar dosage of 0.75 g/L.

To study the desorption of biochar, NaOH, HCl, and CH₃OH solutions were applied, respectively. To study the regenerability of biochar, NaOH, the hot alkaline process, and UV/H_2_O_2_ regeneration methods were applied, respectively. Details of the procedures were provided in Supplementary Text S2.

### 2.5 Calculation and model fitting

The adsorption capacity (*q*_*e*_, mg/g) was determined using Eq. 1:

$$q_{e}=\frac{\left(c_{0}-c_{e}\right)V}{M}$$ (1)

where *c*_*0*_ (mg/L) is the initial TC concentration, *c*_*e*_ (mg/L) is TC concentration at equilibrium, *V* (L) is the solution volume, *M* (g) is the biochar mass, respectively.

The removal rate (*R*, %) was determined using Eq. 2:

$$R=\frac{c_{0}-c_{e}}{c_{0}}\times 100\%$$ (2)

The desorption capacity (*q*_*d*_, mg/g) was determined using Eq. 3:

$$q_{d}=\frac{c_{d}\times V_{d}}{M}$$ (3)

where *c*_*d*_ (mg/L) and *V*_*d*_ (L) are the desorbed TC concentration and the volume of solution, respectively.

The experimental results were fitted using kinetic models (pseudo-first-order, pseudo-second-order, and intraparticle diffusion), and isotherm models (Langmuir and Freundlich), respectively. Supplementary Table S1 presents the expressions for the models used.

## 3 Results and discussion

### 3.1 Characterization

#### 3.1.1 Characterization of PR

The characteristic properties of PR are shown in Table 1. Through the component analysis, PR contained a large number of organic components, such as protein substances (crude protein, 49.52%) and sugar substances (total sugar, 9.28%). As for industrial analysis, the highest content of PR was volatile, 69.40%, and fixed carbon was next, 12.59%. The high volatile content is beneficial to the resource utilization of the bacterial residue, and the higher the content of fixed carbon, the better the preparation of biochar (Wei et al., 2022). The ash content in PR was only 7.21%, indicating that PR contained a few inorganic substances. In addition, there was 10.80% moisture content, which belonged to the combined water in the PR.

**TABLE 1:** Characteristic properties of PR.

Sample	Industrial analysis				Elemental analysis					Component analysis		
	Volatile (%)	Ash (%)	Moisture (%)	Fixed carbon (%)	C (%)	H (%)	O (%)	N (%)	S (%)	Crude protein (%)	Total sugar (%)	Other (%)
PR	69.40	7.21	10.80	12.59	40.61	5.67	30.14	9.36	2.35	49.52	9.28	41.2

The elemental analysis of PR is shown in Table 1. C, H, O, N, and S accounted for 40.61, 5.67, 30.14, 9.36, and 2.35%, respectively. The high content of C and O reflected that PR was mainly composed of organic components. The nitrogen-containing functional groups generated by N elemental during pyrolysis were beneficial to adsorption (Wan and Li, 2018).

Thermogravimetric analysis revealed changes in the thermal stability of PR, and the results are shown in Supplementary Figure S1. Three stages could be distinguished in PR weight loss. The first stage (23–135°C) was the heating dehydration stage. The second stage (135–545°C) was the stage with the largest weight loss. Within this temperature range, a large number of volatile substances were separated out, and organic substances such as fat and protein were pyrolyzed into carbon and produced small molecule gases such as CO and CH_4_ (Guo et al., 2019). The third stage (545–805°C), which was the dehydrogenation and denitrification of organic matter and the continuous condensation stage, the TG curve tended to be gentle, meaning the mass reduction of PR became slow and tended to be stable, and continued warming will cause the collapse of the biochar pore structure (Jiang et al., 2012). According to the thermogravimetric curve, 600°C was selected as the subsequent pyrolysis temperature.

#### 3.1.2 Characterization of biochar

Table 2 shows the TC adsorption capacities of the five kinds of prepared biochars. The adsorption capacity of IKBCH was the highest (267.96 mg/g), followed by that of DKBCH (86.68 mg/g). The adsorption capacities of IKBC, DKBC and BC were 18.49, 14.93, and 5.10 mg/g, respectively, that were far lower than the adsorption capacities of IKBCH and DKBCH.

**TABLE 2:** TC adsorption capacity and textural properties of biochars.

Sample	TC adsorption capacity (mg/g)	BET surface area (m^2^/g)	Micropore area (m^2^/g)	Pore volume (cm^3^/g)	Average pore size (nm)
BC	5.10	0.8821	0.3129	0.0002	0.9851
IKBC	18.49	2.2216	0.4831	0.0045	8.0847
DKBC	14.93	0.8328	0.1367	0.0001	14.8742
IKBCH	267.96	991.3948	901.4568	0.4356	1.7575
DKBCH	86.68	617.5091	489.2215	0.5056	3.2753

According to the structural characteristics of the five biochars listed in Table 2, IKBCH was the microporous biochar with the largest specific surface area (991.3948 m^2^/g) and microporous specific surface area (901.4568 m^2^/g), its average pore diameter was 1.7575 nm, and its adsorption capacity (267.96 mg/g) was the largest among the five biochars. DKBCH was the second one (86.68 mg/g), and its specific surface area (617.5091 m^2^/g) and micropore specific surface area (489.2215 m^2^/g) were reduced compared with IKBCH. Its average pore diameter was 3.2753 nm, which was mesoporous biochar. The specific surface area and micropore specific surface area of BC, DKBC, and IKBC were small (less than 3 m^2^/g), and their adsorption capacities were also small (less than 19 mg/g). Therefore, it could be inferred that the adsorption capacity of TC was depend on the microporous structure and total specific surface area of biochar.

The BET surface area and pore structure characterization showed that, depending on the preparation method, the biochar without acid soaking, including BC, IKBC, and DKBC, had a small specific surface area. This was due to the raw material properties of penicillin residue, the most of the pore channels of the biochar prepared by pyrolysis were blocked, and acid soaking was then needed to unblock these pore channels. The pore size was significantly affected by the activation method, and the pore size of DKBC and IKBC increased significantly compared to that of BC after K_2_CO_3_ activation. In addition, the pore size of biochar prepared by the dry mixing method (DKBC and DKBCH) was larger than that of biochar prepared by the impregnation method (IKBC and IKBCH). The average pore sizes of DKBC, IKBC, DKBCH, and IKBCH were 14.8742, 8.0847, 3.2753, and 1.7575 nm, respectively. Therefore, using different activation methods combined with acid soaking to unblock the occluded pore channels, the mesoporous or microporous biochar could be obtained in a targeted manner.

The K_2_CO_3_ would promote the formation of pore structure. That was because of the pore-forming effect of the gas generated by K_2_CO_3_ during the pyrolysis process, as shown in Eqs 4–7 for the specific reactions. The surface area and microporous structure of IKBCH prepared by the impregnation method were larger than that of DKBCH prepared by the dry mixing method, which was due to the full contact of K_2_CO_3_ with PR in the solution in molecular form, whereas the dry mixing method only made contact on the physical surface. Sufficient activation made subsequent pyrolysis to produce the gas lead to more microporous structures, while acid soaking made the blockage between the pore channels of the biochar completely unblocked. Thus, for the preparation of biochar from PR as raw materials, both activation and acid soaking were necessary steps. Therefore, the biochar IKBCH prepared by activation-pyrolysis-acid soaking had a much higher specific surface area than the biochar prepared by the direct pyrolysis method or the activated-pyrolysis method. Besides, IKBCH was a microporous biochar, while DKBCH was a mesoporous biochar. Related studies reported that mesoporous biochar was more suitable for applications such as carbon-based carrier (Shaikh et al., 2021), while microporous biochar was more suitable for the adsorption of pollutants in water bodies (Wang et al., 2018).

$$K_{2}{\text{CO}}_{3}\to K_{2}O+{\text{CO}}_{2}$$ (4)

$$C+{\text{CO}}_{2}\to 2\text{CO}$$ (5)

$$K_{2}{\text{CO}}_{3}+2C\to 2K+3\text{CO}$$ (6)

$$K_{2}O+C\to 2K+\text{CO}$$ (7)

**FIGURE 1:**
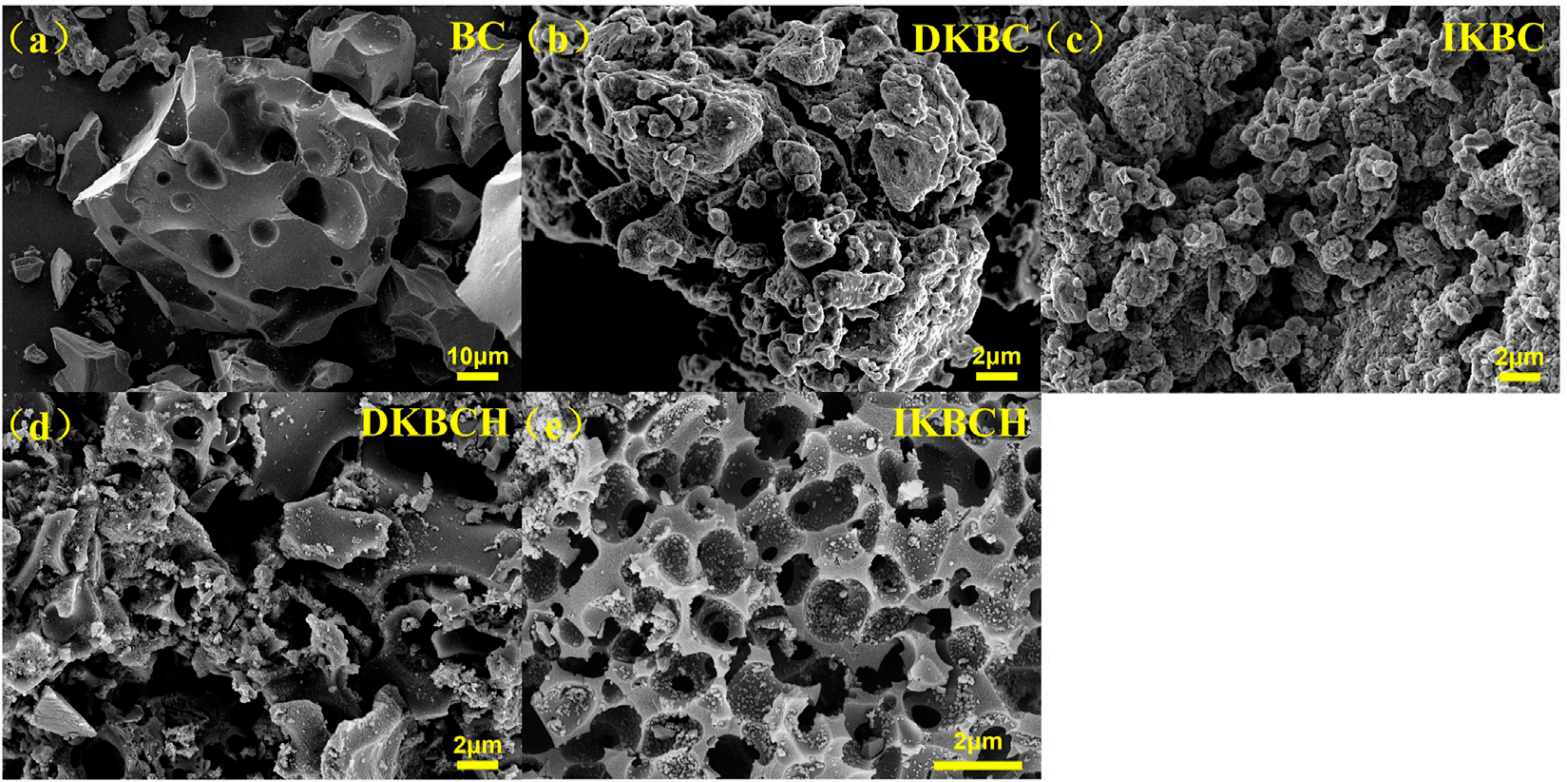
SEM micrographs of **(A)** BC; **(B)** DKBC; **(C)** IKBC; **(D)** DKBCH; **(E)** IKBCH.

Figure 1 shows the SEM characterization results of the five biochars. The surface of BC was relatively flat and distributed with macropore structures. In contrast, DKBC and IKBC had rugged surfaces with swollen folds, that might be attributed to the hollow state inside the biochar caused by gas generated during K_2_CO_3_ pyrolysis. It could be seen that K_2_CO_3_ activation produced great changes to the biochar morphology. In addition, the pore structures of DKBCH and IKBCH were sufficiently loosened after acid soaking so that the surfaces were porous, and according to the SEM results, it was observed that IKBCH had a more regular porous structure, while DKBCH had a more disordered pore structure.

**FIGURE 2:**
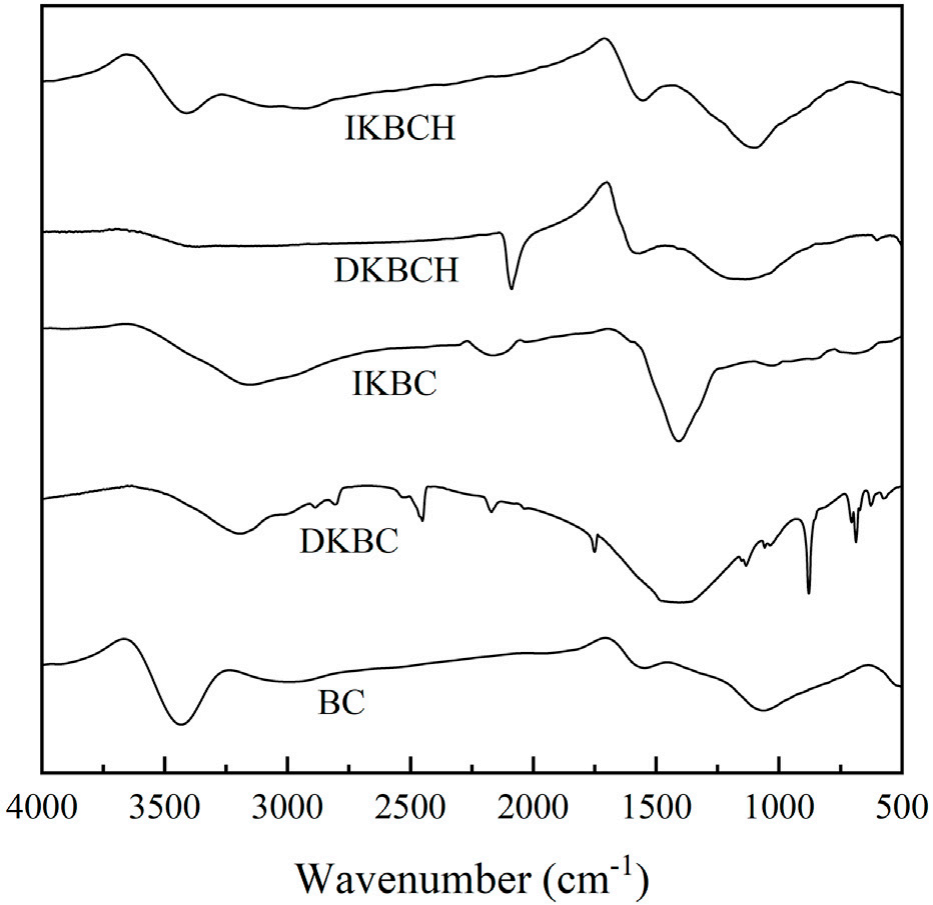
FT-IR spectra of biochars.

Figure 2 shows the FT-IR characterization results of the five biochars. BC showed absorption peaks at 3,439, 1,555, and 1,070 cm^−1^, corresponding to -OH stretching vibration, aromatic C-C stretching vibration and C-O bending vibration, respectively, which were consistent with the general properties of biochar (Dai et al., 2020b; Ma et al., 2021). After being activated by K_2_CO_3_, the -OH absorption peak of IKBC and DKBC were shifted to 3,183 cm^−1^, which might be the combined result of the enhanced C=C-H absorption peak (Qin et al., 2022). The absorption peaks of IKBC and DKBC at 1,417 cm^−1^ were significantly enhanced, similarly attributed to the C-H groups (Zhang et al., 2020). In addition, DKBC showed absorption peaks at 878 cm^−1^ and 588 cm^−1^, corresponding to the C-H group (Hoslett et al., 2021) and the benzene derivatives or aromatic and polysaccharide contents in biochar (Zhang et al., 2020). After acid soaking, the -OH absorption peak and other peaks of DKBCH were weakened, and IKBCH had a similar peak pattern to the BC absorption peak. However, the -OH absorption peak of IKBCH was weakened and the absorption peaks at 1,555 and 1,100 cm^−1^ were strengthened, indicating that the aromatization of IKBCH was strengthened compared with that of BC.

Based on the comprehensive consideration of the above characterization results and TC adsorption capacity, the IKBCH was selected as the study object for TC removal and subsequent experiments were then made to evaluate the adsorption performance of IKBCH.

### 3.2 Effect of dosage on tetracycline removal

**FIGURE 3:**
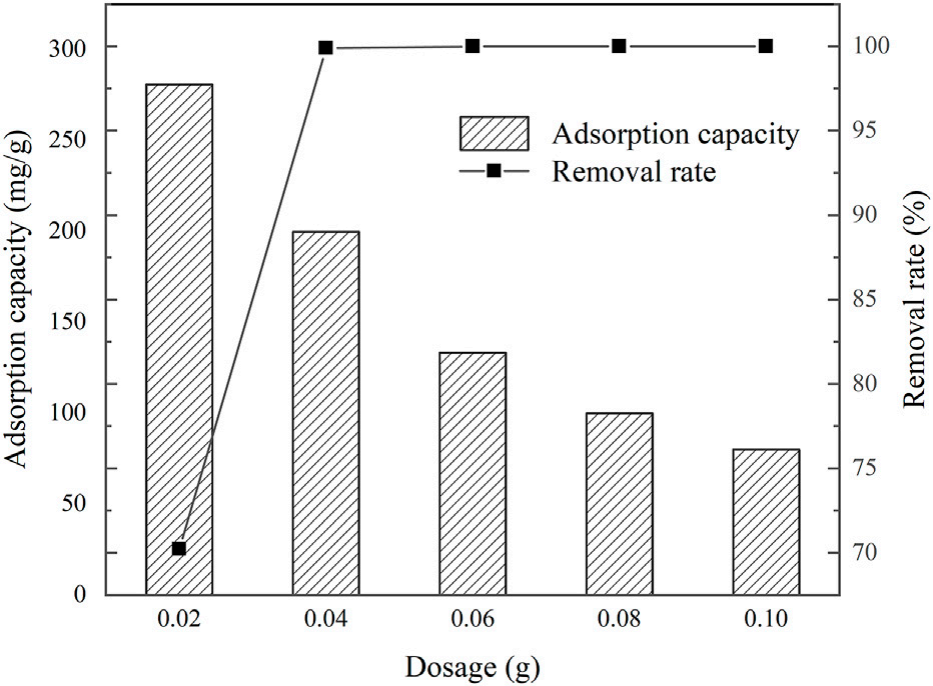
Effect of IKBCH dosing on TC adsorption and removal rate.

The removal efficiency of TC by IKBCH was investigated, and the results are shown in Figure 3. It was observed that a removal rate of 99.91% could be achieved at an initial TC concentration of 200 mg/L with a dosage of 1 g/L. The TC could be completely removed by continuing to increase the dosage of IKBCH. The adsorption capacity of IKBCH decreased with the increase in dosage, it had a maximum adsorption capacity of about 280.91 mg/g at the dosage of 0.5 g/L, and then the adsorption capacity started to decrease due to the insufficient TC concentration, while the removal rate did not decrease. It showed the efficient removal rate of TC by IKBCH.

Table 3 lists the adsorbents prepared by other papers. HMC-800 was a biochar prepared from batatas, and its adsorption capacity for tetracycline was slightly lower than that of IKBCH, but its adsorption equilibrium time was twice as long as that of IKBCH. ZVI@biochar reached adsorption equilibrium at 40 min and also showed a high removal rate in culture wastewater, but its adsorption capacity was only 39.1 mg/g, which was much lower than that of IKBCH. Zn-LBC and BC800 needed more time to reach adsorption equilibrium, probably due to their raw material characteristics, and their removal rates were lower than those of IKBCH. There was still a risk of tetracycline discharge when they were used for tetracycline wastewater removal. In comparison with other adsorbents, IKBCH showed a fast reaction rate, a high adsorption capacity, and was able to achieve complete removal of TC at dosages greater than 1 g/L, showing a better adsorption effect than the previously studied adsorbents.

**TABLE 3:** Comparison of TC adsorption performance of different biochar.

Sample	Raw material	Adsorption capacity (mg/g)	Removal efficiency	Adsorption equilibrium time	References
IKBCH	penicillin fermentation residue	268.6 (25°C)	TC concentration = 200 mg/L, removal rate = 99.91%	2 h	This study
HMC-800	Batatas	238.7 (35°C)	TC concentration = 20 mg/L, removal rate approaching 100%	240 min	Zheng et al. (2021a)
ZVI@biochar	lignocellulosic hazelnut shell	39.1 (298K)	The removal efficiency up to 95% from the culture wastewaters	40 min	Hao et al. (2021)
Zn-LBC	Fraxinus pennsylvanica Marsh leaves	159.6 (298K)	TC concentration = 50 mg/L, removal rate = 71.48%	12 h	Wang et al. (2021c)
BC800	fiberboard biomass	6.37 (298K)	TC concentration = 20 mg/L, removal rate = 68.6%	48 h	Xu et al. (2020)

### 3.3 Effects of initial pH and ionic strength

**FIGURE 4:**
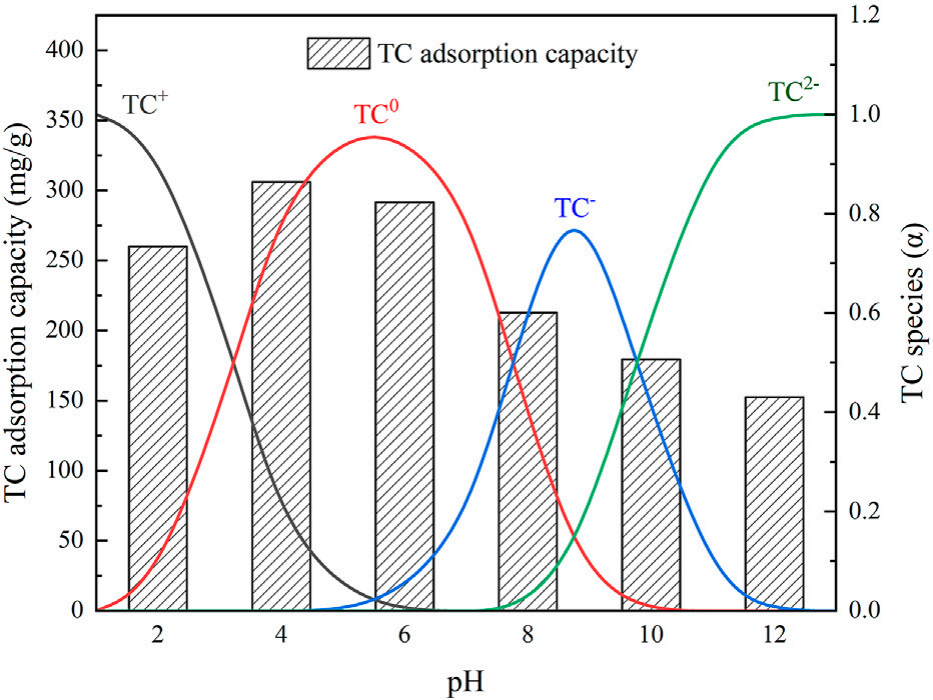
The effect of initial pH on TC adsorption capacity of IKBCH.

The effect of pH on TC adsorption is shown in Figure 4, where it could be seen that the TC adsorption capacity of IKBCH was significantly changed with pH. This is because TC is an amphoteric molecule with pK_a_ values of 3.3, 7.7, or 9.7, and shows different forms in different pH solutions. The main forms of TC at pH < 3.3, 3.3 < pH < 7.7, 7.7 < pH < 9.7, and pH > 9.7 are TC^+^, TC^0^, TC^−^, and TC^2−^, respectively (Kim et al., 2020). The measured pH_pzc_ of IKBCH was 5.1, when pH < 5.1, IKBCH was positively charged, and when pH > 5.1, IKBCH was negatively charged. Owing to the electrostatic interaction, the adsorption capacity of TC by IKBCH was maximum around pH 4–6. The experimental measurement of IKBCH adsorption capacity on TC was 306.00 mg/g at pH = 4. When pH < 3.3, or pH > 7.7, the adsorption capacity of IKBCH decreased significantly. It is noticeable that the minimum adsorption of TC (pH = 12) still maintained 49.8% of the maximum adsorption (pH = 4) under the condition of strong electrostatic repulsion, indicating that other adsorption mechanisms were still present and electrostatic adsorption was not the dominant mechanism. The detailed mechanism analysis is shown in Section 3.6.

**FIGURE 5:**
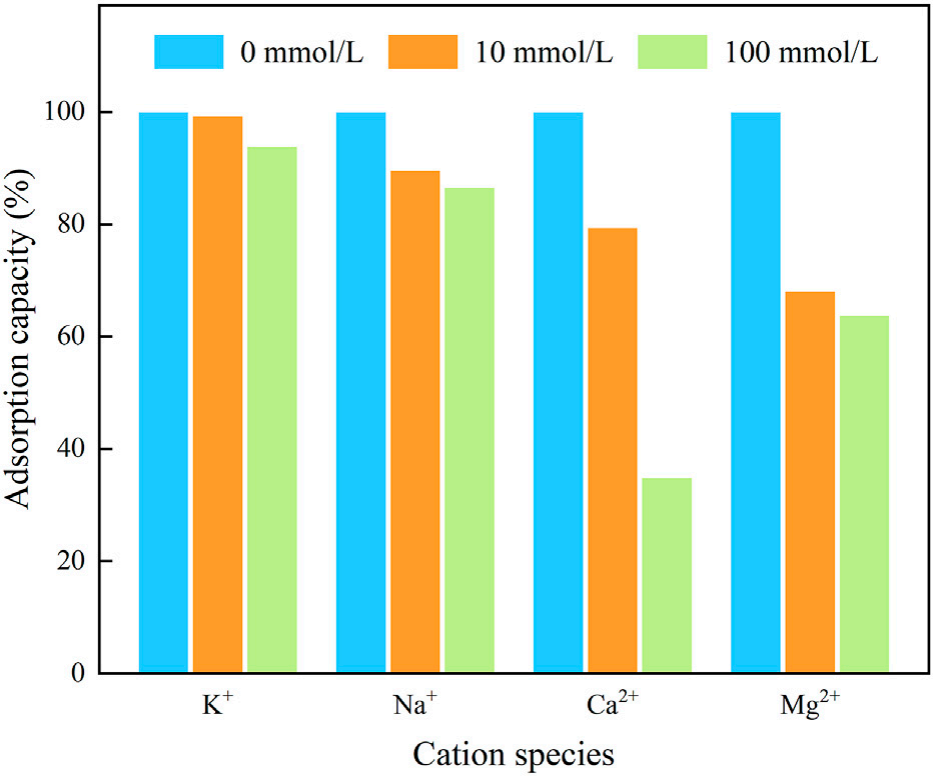
Effect of coexisting cations on TC adsorption capacity of IKBCH.

Considering the presence of cations in the actual waters, which may have an effect on the adsorption process, the effects of several cations, i.e., Na^+^, K^+^, Ca^2+^, and Mg^2+^, on the adsorption of IKBCH on TC were investigated, and the results are shown in Figure 5. Among them, K^+^ had less effect on TC adsorption and was inhibited only at high concentrations (100 mmol/L). The inhibition of TC adsorption by Na^+^ was also within 15%. In contrast, Ca^2+^ and Mg^2+^ caused severe interference, probably due to the fact that divalent cations had higher ionic strengths and therefore had a stronger salting effect than squeezing effect in solution. The higher covalent nature of divalent cations also reduced the number of available adsorption sites on the adsorbent surface. This phenomenon was confirmed in Zhao’s study (Zhao et al., 2021).

### 3.4 Adsorption performance

#### 3.4.1 Adsorption kinetics

**FIGURE 6:**
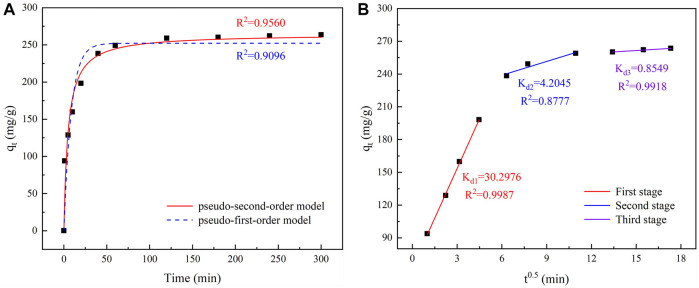
Kinetic curve of TC adsorption and diffusion: **(A)** Fitting curve of adsorption kinetics; **(B)** Fitting curve of Intraparticle diffusion.

To describe the behavior of the adsorption kinetics of TC on IKBCH, the experimental data were fitted using a pseudo-first-order model and a pseudo-second-order model, respectively, and the fitted results are shown in Figure 6A. A rough division of the adsorption process into three stages is shown in Figure 6A. At 0–40 min, the adsorption rate proceeded rapidly, and 90.5% of the equilibrium adsorption capacity was achieved in 40 min. It was related to the fact that IKBCH had sufficient reaction sites at the beginning of the reaction and that the high concentration of TC generated a powerful driving force to overcome resistance to mass transfer. Subsequently, the adsorption reaction slowed down at 40–120 min and reached adsorption equilibrium at 120 min, indicating that the adsorption sites of IKBCH were gradually occupied. At 120–300 min, the reaction was in adsorption equilibrium, and the adsorption sites of IKBCH reached saturation and the adsorption capacity stopped changing. The whole adsorption process had a high correlation coefficient with the pseudo-second-order model (*R* ^2^ = 0.9560), and the theoretical value of adsorption capacity (264.59 mg/g) was very close to the actual measured value (263.49 mg/g). The proposed pseudo-second-order model could reflect TC adsorption behavior on IKBCH, revealing that the adsorption process was chemisorption and could include mechanisms such as hydrogen bonds or π-π interaction (Zheng et al., 2021a).

Based on the experimental data, an intraparticle diffusion model was used to determine possible rate-limiting steps in the adsorption process, and the fitting results are shown in Figure 6B. There were generally three stages in controlling the adsorption rate and mechanism of adsorbents, surface diffusion, which indicated that the adsorbent crossed the phase interface resistance to reach the adsorbent surface, inner diffusion, which indicated that the absorbates entered the internal active sites and pores from the adsorbent surface, and equilibrium stage, which was the stage where adsorption reached equilibrium (Chen et al., 2021). As described in Figure 6B, the fitted curve did not pass through the origin, indicating that the rate-limiting steps were controlled by both surface diffusion and inner diffusion, and that K_d1_ = 30.2976 was much higher than the other two stages, indicating that surface diffusion was the main rate-limiting step for the whole process (Qin et al., 2022).

#### 3.4.2 Adsorption isotherms

**FIGURE 7:**
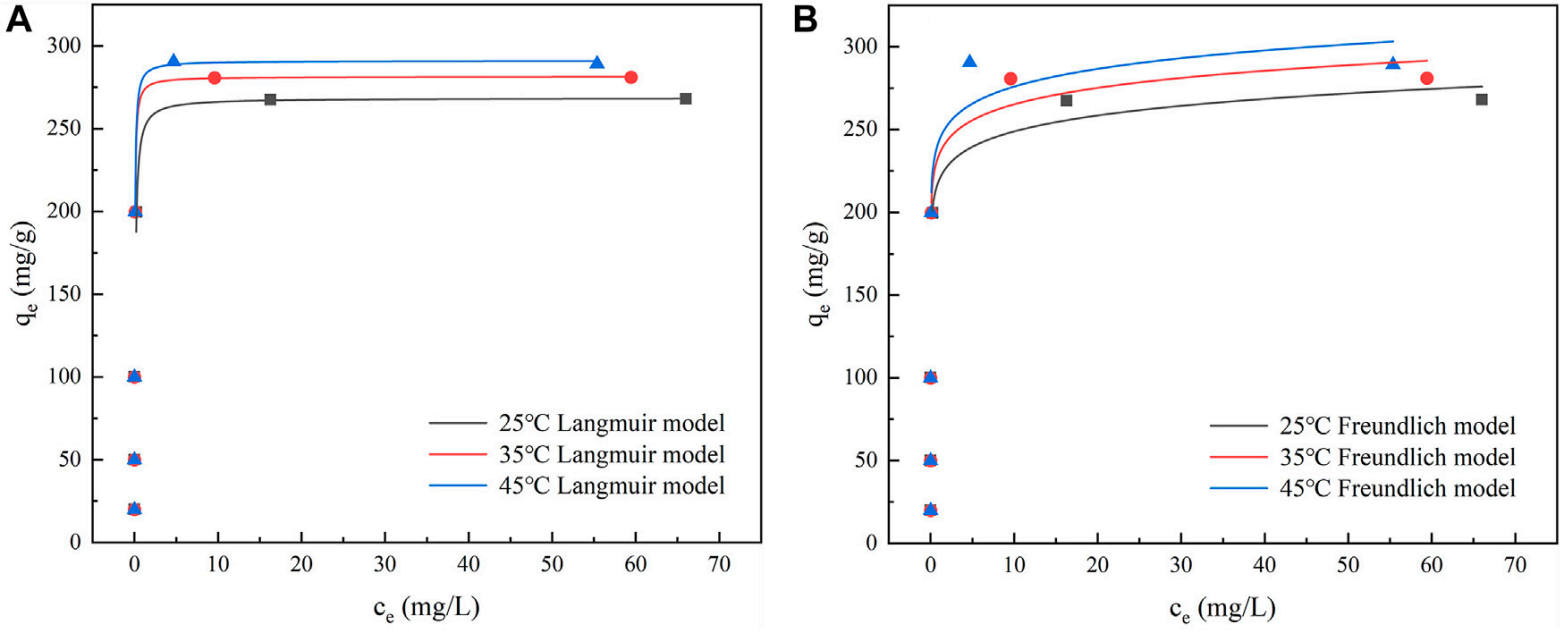
Fitting curve of TC adsorption isotherm: **(A)** Langmuir model; **(B)** Freundlich model.

The adsorption behavior of IKBCH at different temperatures and different TC concentrations was shown in Figure 7 and Supplementary Table S2. It was observed that TC adsorption increased with increasing TC concentration and temperature. The Langmuir model and Freundlich model were then used to fit the experimental data, respectively. It was found that the Langmuir model could well describe the adsorption behavior of TC on IKBCH (*R* ^2^ > 0.997), and the theoretical maximum adsorption at 25, 35, and 45°C were 268.55, 281.54, and 291.18 mg/g, respectively, and they all were almost consistent with the actual experimental values. In general, the Langmuir isotherm model assumes that adsorption sites on the adsorbent surface are homogeneous and energy-equivalent, and are bound to the adsorbent on a monolayer. Once the site was taken up by an adsorbed molecule, the site cannot be further adsorbed (Zheng et al., 2021b). Whereas the Freundlich isotherm model has an assumption about the non-homogeneity of the adsorbent surface, which is applicable to multilayer adsorption layers (Koh et al., 2022). The correlation coefficient of IKBCH to the Langmuir model (*R* ^2^ > 0.997) exceeds that of the Freundlich model (R > 0.626), indicating that the adsorption of TC on IKBCH occurred on the monolayer.

### 3.5 Desorption and recycle performance

The desorption ability of IKBCH is shown in Supplementary Table S3. NaOH had the best desorption ability (about 33%), and both HCl and methanol had less than 5%, indicating that the adsorption of TC was relatively stable and would not cause pollution in the natural aqueous environment. IKBCH has a relatively stable adsorption performance and can be applied as an ideal adsorbent. However, the stability of IKBCH under the interference of physical, chemical and microbial processes needs to be further investigated.

The cycling performance of IKBCH is shown in Supplementary Figure S2. The best regeneration method was the hot alkali method, followed by the UV/H_2_O_2_ method. The NaOH method was less effective than the other two methods. After three cycles, the adsorption capacity of IKBCH decreased to 192.13, 75.02, and 47.98 mg/g, by the hot alkali method, the UV/H_2_O_2_ method, and the NaOH method, respectively, suggesting that the adsorption site of IKBCH could not be reversed entirely. Although the adsorption performance of IKBCH decreased after regeneration, its adsorption capacity remained applicable to the actual wastewater treatment.

### 3.6 Adsorption mechanism

According to previous studies, pore filling was one of the important mechanisms of TC adsorption (Li et al., 2021a). Based on the BET results and the adsorption capacity results in Table 2, it could be seen that the specific surface area is directly correlated with TC adsorption capacity. The IKBCH, prepared by the impregnation-pyrolysis-acid soaking method, was the microporous biochar with the largest specific surface area, and the TC adsorption capacity of the IKBCH far exceeded that of other biochars. The TC molecule size was reported to be about 1.27 nm (Qin et al., 2022), and the TC molecule could easily enter inside the pores of IKBCH and make full use of the microporous structure as an adsorption site. The intraparticle diffusion model also indicated that the diffusion of TC molecules in the internal pores controlled the adsorption process. It was inferred that pore filling was one of the important mechanisms for TC adsorption by IKBCH.

**FIGURE 8:**
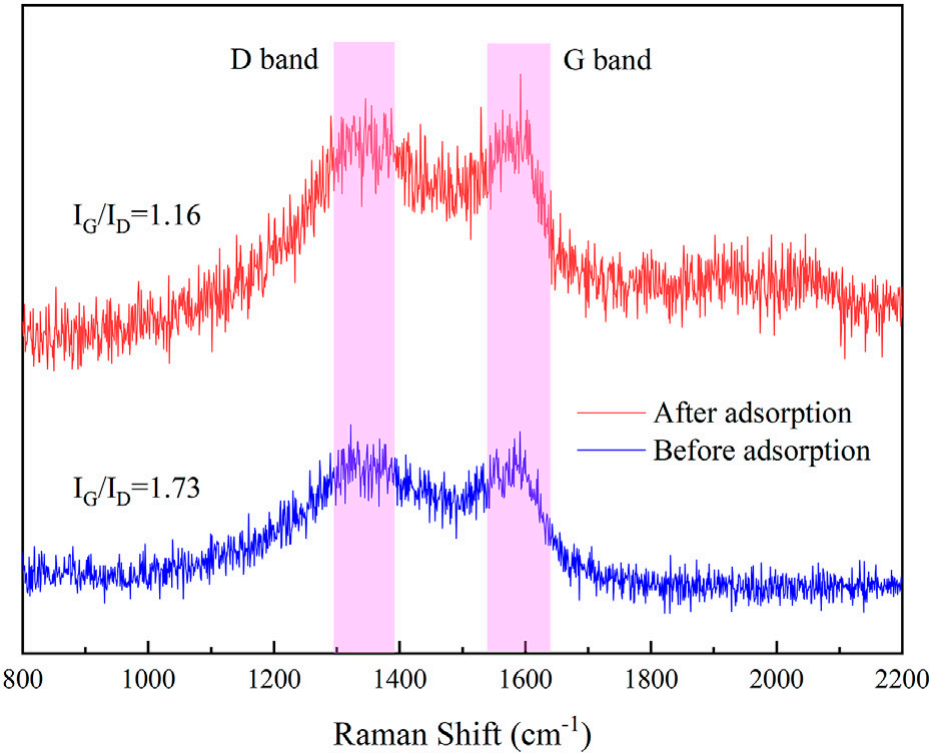
Raman spectra of IKBCH before and after TC adsorption.

The Raman spectra of IKBCH are shown in Figure 8. Two characteristic peaks had been observed, corresponding to the D-band (1,350 cm^−1^) and the G-band (1,585 cm^−1^). The D-band suggested that the carbon lattice was disordered, and the defective ones were in the form of amorphous carbon. The G-band indicated the in-plane tangential stretch of the ordered sp2 bonded carbon (Liu et al., 2020). The higher the I_D_/I_G_ value, the higher the disorder and defectivity of the carbon material. The π-electrons of the IKBCH graphene layer would interact with the π-electrons of the TC aromatic ring in a π-π interaction. After adsorption, the I_D_/I_G_ values of IKBCH decreased from 1.73 to 1.16, confirming that π-π interactions were a significant mechanism for TC adsorption by IKBCH (Li et al., 2021a).

**FIGURE 9:**
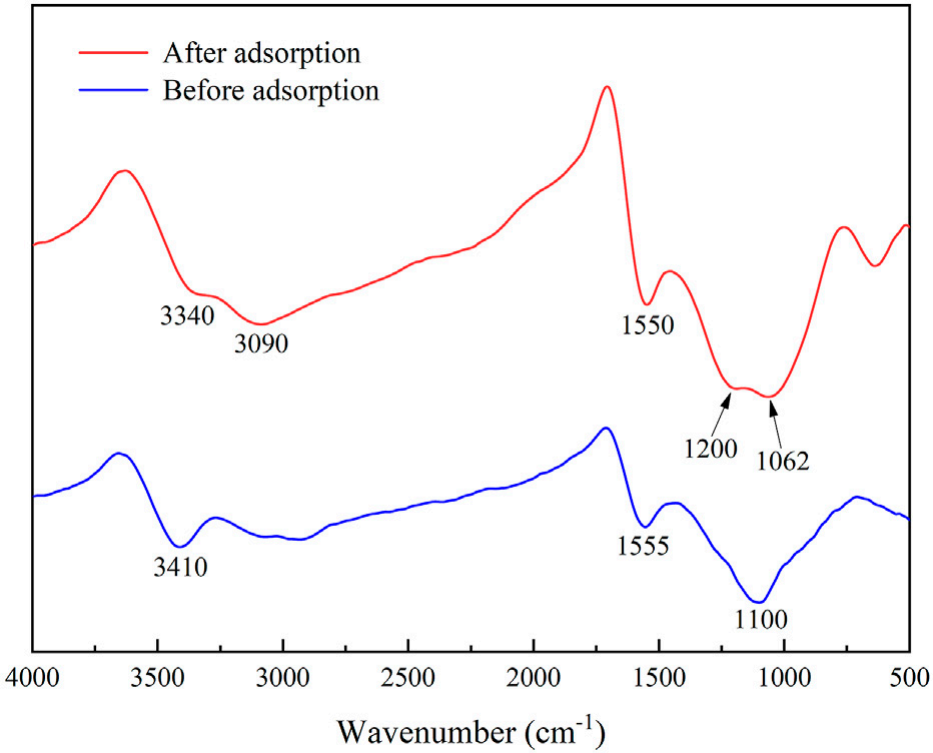
FT-IR spectra of IKBCH before and after TC adsorption.

In the removal of TC, biochar’s functional groups might play an important role. The FT-IR results of IKBCH are shown in Figure 9. Before adsorption, three major absorption peaks were at 3,410, 1,555, and 1,100 cm^−1^. The aromatic structure and oxygen-containing functional groups could be bound to aromatic organic pollutants through hydrogen bonding as π-electron acceptors (Chen et al., 2021). After TC adsorption, the hydroxyl absorption peak was weakened, and the C-O absorption peak was shifted from 1,100 to 1,062 cm^−1^, indicating that the oxygen-containing functional group was involved in the adsorption reaction (Li et al., 2021a; Chen et al., 2021). The presence of a new absorption peak at 3,090 cm^−1^ might be caused by the stretching vibration of the C=C-H group in the TC molecule. The absorption peak at 1,200 cm^−1^ was the C-N stretching vibration. The results proved that TC adsorption by IKBCH was through chemical bonding (Qin et al., 2022).

**FIGURE 10:**
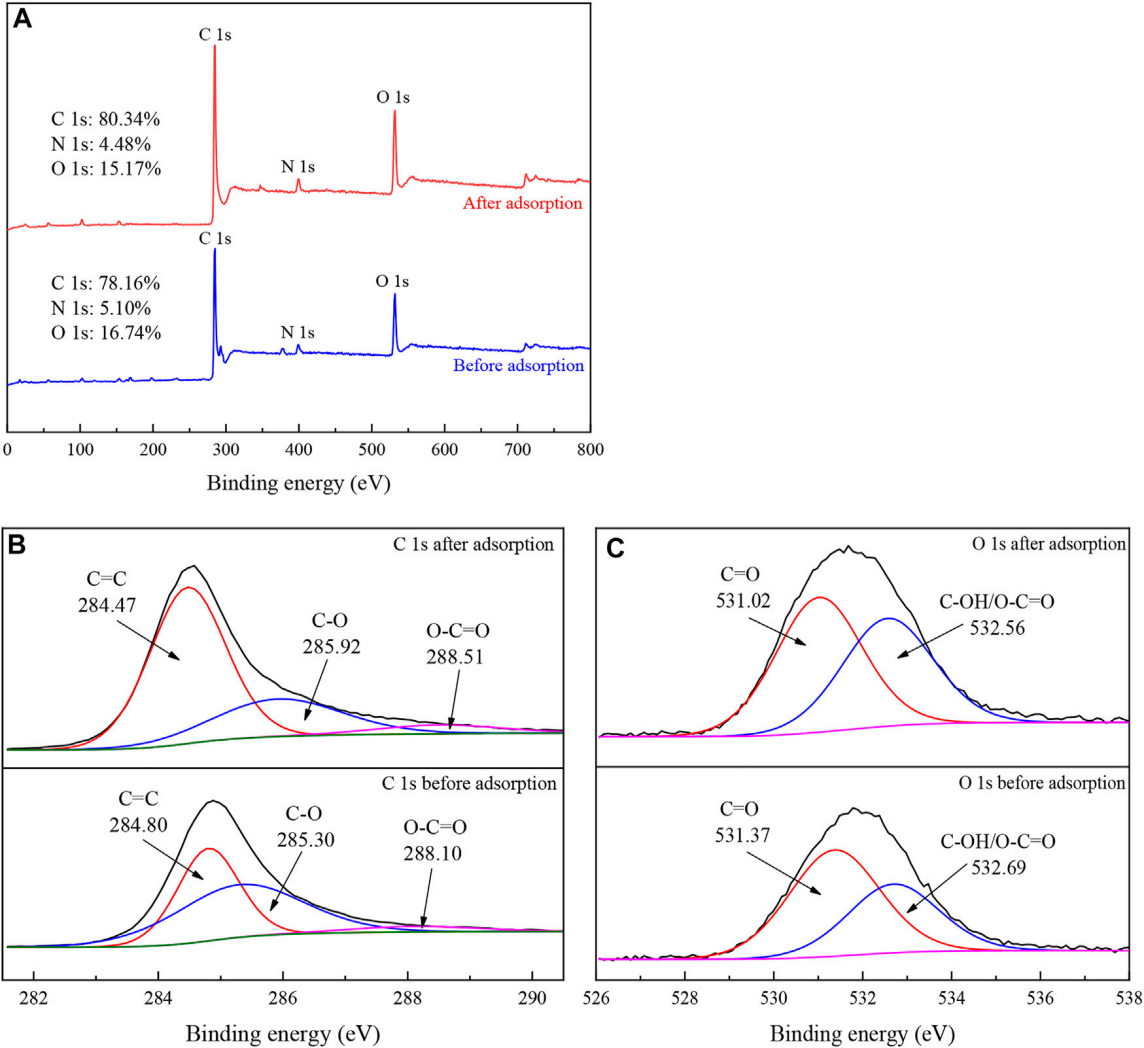
XPS spectra of IKBCH before and after TC adsorption: **(A)** Survey spectra; **(B)** C1s spectra; **(C)** O1s spectra.

According to the XPS results shown in Figure 10, the contents of C, N, and O changed after adsorption, indicating that TC was successfully adsorbed. The C1s spectrum showed that the content and binding energy of C=C (284.80 eV), C-O (285.30 eV) and O-C=O (288.10 eV) changed, and the increase of C=C and O-C=O and the decrease of C-O indicated that the π-π interaction between the aromatic ring in TC molecule and the aromatic carbon part in IKBCH was involved in the adsorption process (Li et al., 2021b; Chen et al., 2021; Yu et al., 2022). According to the O1s spectrum, the contents of C=O (531.37 eV) and C-OH/O-C=O (532.69 eV) changed, indicating that the interaction of TC molecules with the C-OH/O-C=O group of IKBCH was through hydrogen bonding or electrostatic attraction (Mu et al., 2021; Yu et al., 2022).

According to the initial pH effect experiments in Section 3.3, the surface charge of IKBCH and the morphology of TC significantly affected the adsorption capacity of IKBCH. The electrostatic interaction was also involved in the TC adsorption by IKBCH.

In the above, it could be seen that IKBCH adsorbed TC mainly depending on the pore structure and fragrant graphite structure, and the functional group structure also had a part to play in the adsorption process. The mechanism of the TC adsorption by IKBCH could be concluded as pore filling, π-π interaction, electrostatic adsorption and hydrogen bonding. The adsorption process can be expressed in Supplementary Figure S3.

### 3.7 Environmental impact and economic feasibility

In the local pharmaceutical industry, the disposal of PR is usually done by incineration, and the cost of this disposal was known to be about RMB 2500/ton. The preliminary calculation of the raw material cost of biochar preparation was about RMB 1052/ton (the cost of pyrolysis was RMB 162/ton, the cost of K_2_CO_3_ was RMB 640/ton, the cost of HCl was RMB 250/ton, other costs were excluded for the time being, and the cost was calculated according to the Chinese market price). It was seen that the preparation of biochar by pyrolysis to treat PR was a very promising scheme that could be applied to practical projects on a large scale.

Related studies have shown that penicillin can be completely decomposed under high temperature pyrolysis (Wang et al., 2021b). The drug residues of penicillin in the prepared biochar were measured, and the results are shown in Supplementary Table S4. The results showed that no antibiotic drug residues were detected. The high-temperature pyrolysis resulted in the complete decomposition of penicillin, confirming that there was no environmental risk in the application of IKBCH for water treatment.

To illustrate that IKBCH was an effective and versatile adsorbent for environmental applications, IKBCH was used to adsorb different organic pollutants (Including dyes and antibiotics). Table 4 shows the adsorption capacity of IKBCH for other pollutants. The results showed that IKBCH had excellent adsorption performance on all types of pollutants. It was confirmed that IKBCH has wide applicability and can be used for the removal of many kinds of pollutants.

**TABLE 4:** Adsorption capacity of IKBCH on other pollutants.

Pollutants	Adsorption capacity (mg/g)
Ranitidine	271.49
Ciprofloxacin	318.68
Rhodamine B	496.45
Methylene blue	349.88
Bisphenol A	319.53
Phenol	89.95

## 4 Conclusion

In this study, biochar with a high specific surface area was prepared by pyrolysis using penicillin fermentation residue as raw material, combined with K_2_CO_3_ activation and hydrochloric acid soaking. According to the activation by the dry mixing method or the impregnation method, mesoporous or microporous biochar could be prepared, among which microporous biochar IKBCH had the best performance on TC adsorption. The pseudo-second-order model and Langmuir isotherm model could better fit the adsorption experimental data, respectively. The main adsorption mechanisms involve pore filling, π-π interaction, hydrogen bonding and electrostatic adsorption. The adsorption of TC by IKBCH was very stable and would not cause secondary pollution in the environment. In addition, IKBCH had good adsorption performance on a variety of pollutants, and it is a promising adsorbent.

## Data Availability

The original contributions presented in the study are included in the article/Supplementary Material, further inquiries can be directed to the corresponding author.
