# Supplementary material for: Efficient removal of tetracycline from aqueous solution by K2CO3 activated penicillin fermentation residue biochar
Source: Front Chem. 2022 Dec 13;10:1078877. doi: 10.3389/fchem.2022.1078877 (PMC9792616; doi:10.3389/fchem.2022.1078877)
Supplement: Supplementary file 1 [file DataSheet1.docx]

Supplementary Material

**Text. S1 Characterizations**

Total sugar content in penicillin fermentation residues (PR) was measured by the phenol-sulfuric acid colorimetric method. Crude protein content in PR was measured by the Kjeldahl method. Crude fat content in PR was measured by the Soxhlet extraction method.

High-performance liquid chromatography-mass spectrometry (HPLC-MS; Agilent 6410B, USA) was used to determine antibiotic residues in biochar.

**Text. S2 Batch experiments**

To study the desorption of biochar, NaOH, HCl, and CH₃OH were applied. 0.03 g of adsorbed saturated biochar sample was added to 40 ml of 1 mol/L solution of HCl, NaOH and CH₃OH, respectively. The flasks were shaken at 25℃ at 200 rpm for 2 h. Then, the samples were filtered through a 0.45 µm membrane and investigated by a spectrophotometer to calculate the desorption amount.

To study the renewability of biochar, the NaOH method, the hot alkaline method, and the UV/H_2_O_2_ regeneration method were applied. NaOH method: 0.03 g of adsorption-saturated biochar sample was placed in 40 mL of NaOH solution with a concentration of 1 mol/L, shaken at 25℃ and 200 rpm for 2 h to complete a regeneration, followed by drying the sample for the next cycle of the adsorption experiment. Hot alkali method: 0.1 g of adsorption-saturated biochar sample was placed in 40 mL of NaOH solution with a concentration of 1 mol/L at 55℃ and shaken at 200 rpm for 2 h to complete a regeneration, followed by drying the sample for the next cycle of the adsorption experiment. UV/H_2_O_2_ regeneration method: 0.1 g of adsorption-saturated biochar sample was placed in 40 mL of H_2_O_2_ (5 wt%) solution, with UV lamp irradiation, and shaken at 25℃ and 200 rpm for 2 h to complete the regeneration, followed by drying the sample for the next cycle of the adsorption experiment.

**Text. S3 Desorption and recycle performance**

The desorption ability of IKBCH was shown in Table S1. The cycling performance of IKBCH was shown in Figure S2.

**Text. S4 Environmental impact and economic feasibility**

Antibiotic residues in biochar measured by HPLC. The results were shown in Table S3.

**Table S1** List of kinetic and isotherm models

| Models | Expressions |
| --- | --- |
| Pseudo-first-order | $q_{t}=q_{e}\left( 1-e^{-k_{1}t} \right)$ |
| Pseudo-second-order | $q_{t}=\frac{q_{e}^{2}k_{2}t}{1+q_{e}k_{2}t}$ |
| Intraparticle diffusion | $q_{t}=k_{di}t^{1/2}+C$ |
| Langmuir | $q_{e}=\frac{q_{m}K_{L}c_{e}}{1+K_{L}c_{e}}$ |
| Freundlich | $q_{e}=K_{F}c_{e}^{1/n}$ |

Where *q_e_* (mg/g) is the adsorbed amount of TC at equilibrium, *q_t_* (mg/g) is the adsorbed amount of TC at time t. *k_1_* is the rate constant for the pseudo-first-order (1/h), *k_2_* is the rate constant for the pseudo-second-order (g/mg·h), and *k_di_* is the rate constant for the intraparticle diffusion (mg/g·h^1/2^) rate constant. *c_e_* (mg/L) is the TC concentration at equilibrium, *q_m_* (mg/g) is the Langmuir maximum capacity, *K_L_* (L/mg) is the Langmuir constant, *K_F_* (mg/g) is the Freundlich adsorption capacity, and n is the adsorption intensity.

**Table S2** Adsorption parameters of TC adsorption isotherm model

| Temperature | Langmuir | | | Freundlich | | |
| --- | --- | --- | --- | --- | --- | --- |
|  | q_m_ | K_L_ | R^2^ | 1/n | K_F_ | R^2^ |
| 25℃ | 268.5516 | 11.7133 | 0.9999 | 0.0549 | 219.4036 | 0.8602 |
| 35℃ | 281.5401 | 29.7432 | 0.9999 | 0.0531 | 234.7635 | 0.8161 |
| 45℃ | 291.1798 | 26.6277 | 0.9978 | 0.0549 | 243.2564 | 0.6262 |

**Table S3** The desorption ability of IKBCH.

| Desorption method | Desorption capacity (mg/g) |
| --- | --- |
| NaOH | 86.02 |
| HCl | 9.84 |
| CH₃OH | 12.43 |

**Table S4** Antibiotic concentration (ng/L).

| Penicillin types | Sample 1 | Sample 2 |
| --- | --- | --- |
| Penicillin G | Undetected | Undetected |
| Amoxicillin | Undetected | Undetected |
| Ampicillin | Undetected | Undetected |
| Cloxacillin | Undetected | Undetected |
| Benazocillin | Undetected | Undetected |
| Dicloxacillin | Undetected | Undetected |
| Cefuroxime | Undetected | Undetected |
| Cefquinoxime | Undetected | Undetected |
| Ceflonin | Undetected | Undetected |
| Cefazolin | Undetected | Undetected |
| Cefoperazone | Undetected | Undetected |
| Cefazolin | Undetected | Undetected |

**Figure S1** TG and DTG curves of PR at heating rate of 10 °C/min

**Figure S2** Effect of cycle time on the TC adsorption capacity of IKBCH


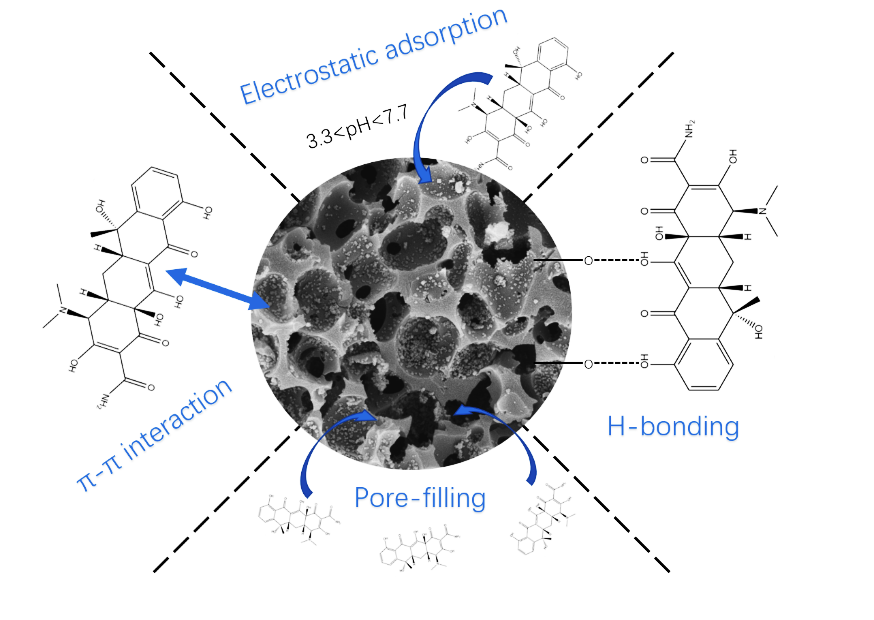


**Figure S3** TC adsorption mechanism schematic diagram
